# Haplotype-resolved assemblies of the MHC region in five widely used tumor cell lines

**DOI:** 10.1016/j.gendis.2025.101603

**Published:** 2025-03-18

**Authors:** Haozhe Yuan, Mengping Jiang, Xingyu Xu, Jialiang Zhu, Shulong Dong, Weida Meng, Dandan Zhang, Jiakang Ma, Yicheng Lin, Ziqiang Chen, Shaoyang Sun, Wenqing Qiu, Yun Liu

**Affiliations:** a MOE Key Laboratory of Metabolism and Molecular Medicine, Department of Biochemistry and Molecular Biology, School of Basic Medical Sciences and Shanghai Xuhui Central Hospital, Fudan University, Shanghai 200032, China; b State Key Laboratory of Medical Neurobiology and MOE Frontiers Center for Brain Science, Institutes of Brain Science, Fudan University, Shanghai 200032, China; c MOE Key Laboratory of Metabolism and Molecular Medicine, Department of Biochemistry and Molecular Biology, School of Basic Medical Sciences, Fudan University, Shanghai 200032, China; d Shanghai Xuhui Central Hospital, Shanghai 200032, China

**Keywords:** Cell lines, CRISPR-Cas9, Haplotype-resolved assembly, MHC, Targeted sequencing

## Abstract

The major histocompatibility complex (MHC) region plays a crucial role in immune function and is implicated in various diseases and cancer immunoediting. However, its high polymorphism poses challenges for accurate genetic profiling using conventional reference genomes. Here, we present high-quality, haplotype-resolved assemblies of the MHC region in five widely used tumor cell lines: A549, HeLa, HepG2, K562, and U2OS. Numerous oncological studies extensively employ these cell lines, ranging from basic molecular research to drug discovery and personalized medicine approaches. By integrating CRISPR-based targeted enrichment with 10 × Genomics linked-read and PacBio HiFi long-read sequencing, we constructed MHC haplotypes for each cell line, providing a valuable resource for the research community. Using these assembled haplotypes as references, we characterize the aneuploidy of the MHC region in these cell lines, offering insights into the genetic landscape of this critical immunological locus. Our work addresses the urgent need for accurate MHC profiling in these widely used cell line models, enabling more precise interpretation of existing and future genomic and epigenomic data. This resource is expected to significantly enhance our understanding of tumor biology, immune responses, and the development of targeted therapies.

## Introduction

The major histocompatibility complex (MHC) region, located on chromosome 6 in humans and spanning approximately 4.5 Mb, harbors genes encoding a diverse array of molecules crucial for antigen presentation. These MHC genes are designated human leukocyte antigen (HLA) genes in humans. MHC class I and II molecules present antigens to CD8^+^ and CD4^+^ T cells, respectively, initiating inflammatory responses against pathogens and malignant cells.^1^ As a critical genomic region for immune function, genetic and epigenetic polymorphisms within the MHC locus are associated with numerous human diseases.^2^^,^^3^ Moreover, somatic alternations and clonal loss of MHC genes are hallmarks of cancer immunoediting, as tumors evolve within an immunocompetent host, thereby impeding immune surveillance.^4^^,^^5^ Consequently, elucidating the genetic architecture of the MHC region and its regulation is pivotal for advancing our understanding of human diseases and cancer, holding substantial clinical prognostic value.

Tumor-derived cell lines serve as indispensable research tools in the study of tumor immunoediting mechanisms. These cell lines enable the modeling of molecular mechanisms underlying tumor biology, yielding valuable insights into tumor growth and therapeutic responses.^6^ Consequently, comprehensive analyses of genome sequences and genetic characteristics of these cell line models are crucial. Recent years have witnessed a focus on the genomic profiling of widely used tumor cell lines through next-generation sequencing, facilitating more accurate interpretation of extensive genomic and epigenomic data generated from these cell line models.^7^, ^8^, ^9^ The advent of long-read sequencing technologies, such as PacBio high-fidelity (HiFi) sequencing and Oxford Nanopore Technologies (ONT) nanopore sequencing, has further advanced the field. Notably, researchers have successfully assembled telomere-to-telomere (T2T) sequences of the homozygous CHM13hTERT cell line, establishing a haplotype-resolved human reference genome.^10^

Given the high polymorphism of the MHC region, genetic profiling of the MHC region in commonly used cell lines becomes even more crucial. The MHC locus stands as one of the most polymorphic regions in the human genome, shaped by positive and balancing selection and further complicated by strong linkage disequilibrium.^11^ Analyses of the MHC region using conventional reference genomes like the GRCh38 can lead to biased read alignment and inaccurate quantifications due to significant divergence between the reference sequence and the highly variable MHC region in cell lines, resulting in inaccurate inferences.^12^^,^^13^ Consequently, the generation of complete, high-quality haplotype-resolved assemblies of the MHC region for commonly used cell lines emerges as both an urgent necessity and a valuable resource for the research community.

In this study, we employed a previously developed approach that integrates CRISPR-based targeted enrichment with 10 × Genomics linked-read and PacBio HiFi long-read sequencing technologies.^14^ This approach enabled us to construct high-quality, haplotype-resolved assemblies of the MHC regions in five widely used tumor cell lines: A549 (lung adenocarcinoma), HeLa (cervical adenocarcinoma), HepG2 (hepatocellular carcinoma), K562 (chronic myelogenous leukemia), and U2OS (osteosarcoma). Utilizing these targeted assembled MHC haplotypes as references, we characterized the aneuploidy of the MHC region in these cell lines, providing a comprehensive genetic landscape of this crucial immunological locus.

## Material and methods

### Cell lines and cell culture

A549 cells (SCSP-503), HeLa cells (SCSP-504), HepG2 cells (SCSP-510), K562 cells (SCSP-5054), and U2OS cells (SCSP-5030) were purchased from the Stem Cell Bank of the Chinese Academy of Sciences. All the cell lines underwent testing for mycoplasma, bacterial endotoxins, bacteria, and viruses in accordance with the quality control procedures established by the NSCRC AMS.^15^ A549, HeLa, U2OS, and HepG2 cells were cultured as adherent monolayers in Dulbecco's modified Eagle medium (Gibco) supplemented with 10% fetal bovine serum (Gibco) and 1 × penicillin-streptomycin (Thermo Fisher Scientific) in cell culture dishes. K562 cells were maintained in suspension in T25 or T75 flasks using RPMI 1640 medium (Gibco) supplemented with 10% fetal bovine serum (Gibco) and 1 × penicillin-streptomycin (Thermo Fisher Scientific). Mycoplasma testing was performed each week.

### Preparation of cell-embedded agarose plugs

The preparation of megabase-sized MHC DNA molecules from cell lines was adapted from a previously established protocol with modifications.^14^ Briefly, harvested cells were washed three times in ice-cold phosphate buffer saline and resuspended to a final concentration of approximately 2 × 10^7^ cells/mL in L buffer (0.1 M EDTA, pH 8.0; 0.01 M Tris-Cl, pH 7.6; and 0.02 M NaCl). The cell suspension was incubated at 42 °C for 5 min. Concurrently, 1.2% low-melting-point agarose was melted at 70 °C, equilibrated at 42 °C for 5 min, and then mixed in a 1:1 ratio with the cell suspension. This mixture was immediately dispensed into a plug mold, with 1 mL of the cell-agarose mixture filling 10 wells (80 μL/well) in the mold. The plugs were then incubated at 4 °C until solidification. Once solidified, the agarose plugs were transferred into a solution of L buffer containing 0.5 mg/mL proteinase K and 1% (w/v) Sarkosyl and incubated at 50 °C for 3 h. The original digestion buffer was then replaced with fresh digestion buffer, and incubation continued for an additional 12–16 h at 50 °C. Following digestion, the plugs were rinsed three times with 50 volumes of TE buffer (10 mM Tris, pH 8.0; and 1 mM EDTA) over 3 h. They were then incubated with TE buffer containing 40 μg/mL phenyl methyl sulfonyl fluoride at room temperature for 1 h, followed by a 30-min incubation at 50 °C. The plugs were washed three more times with TE buffer over 3 h and were either subjected to Cas9 digestion or stored in TE buffer at 4 °C for future use.

### Design and generation of sgRNAs

**Figure 1:**
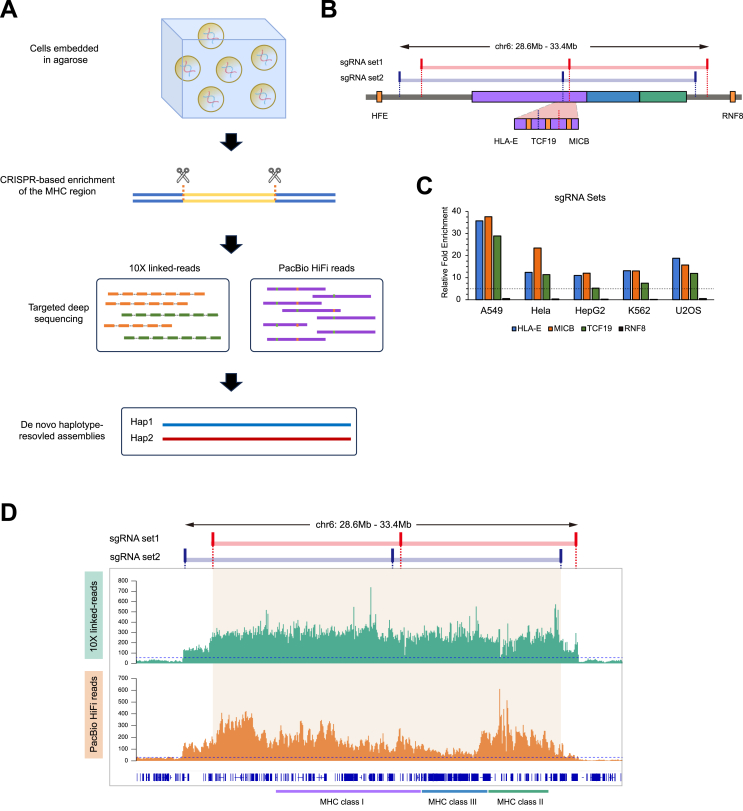
Targeted enrichment of the MHC region of tumor cell lines. **(A)** A detailed diagram illustrating the CRISPR-based targeted haplotype-resolved assembly of the MHC region. **(B)** The positions of two sets of sgRNAs targeting the MHC region. The lines and boxes colored with semitransparent blue and red represent two sets of sgRNAs showing the targeting sites, respectively. The targeted region spans from 28.6 Mb to 33.4 Mb, while the violet, blue, and green colored boxes represent the regions encompassing HLA class I, class III, and class II genes, respectively. The amber boxes denote the genes used for quantitative real-time PCR analyses, listed according to their coordinates on the GRCh38 reference. **(C)** The relative enrichment of the targeted MHC region of the tumor cell lines was validated using quantitative real-time PCR. The fold enrichment was quantified relative to the *HFE* gene, located upstream of the targeted region, and normalized to cells treated without sgRNAs. The relative enrichment for *RNF8*, a gene outside the targeted MHC region, was tested as a negative control. Data were obtained from two independent experiments. **(D)** The sequencing coverage of the targeted MHC region of the A549 cell line based on data from the 10× Genomic linked-read and PacBio HiFi sequencing platforms. The beige area, encompassed by the two sets of sgRNAs (red and blue bars at the top), highlights the targeted MHC region. The dashed lines indicate a 50-fold enrichment for 10× Genomics linked-read data (green) and a 30-fold enrichment for PacBio HiFi data (orange).

Two sets of sgRNAs with 20-bp sequences (20-mers) were designed from non-polymorphic genomic regions flanking the targeted MHC region. Set 1 spanned chr6: 28.90–33.46 Mb, and set 2 covered chr6: 28.58–33.27 Mb, as depicted in Figure 1B. All sgRNA sequences were selected using the CRISPRdirect tool (https://crispr.dbcls.jp). The sgRNAs were synthesized *in vitro* using the EnGen sgRNA Synthesis Kit (NEB) and subsequently purified with the RNA Clean & Concentrato-25 kit (Zymo Research). The purified sgRNAs were then quantified using a NanoDrop spectrophotometer (Thermo Fisher Scientific) to ensure accurate concentration measurements for downstream applications.

### CRISPR-based cleavage of the targeted MHC region

Agarose plugs containing cells were digested *in vitro* using the S. pyogenes Cas9 nuclease (NEB), following a previously described method with modifications.^14^ Specifically, sgRNAs and Cas9 enzyme were pre-assembled prior to digestion by mixing 4 pmol of Cas9 enzyme with 150 ng of sgRNAs, 6 μL of 10 × Cas9 buffer, 1U/μL RNasin ribonuclease inhibitor, and nuclease-free water to a final volume of 60 μL. This mixture was incubated at 37 °C for 15 min. The cleavage efficiency of each pre-assembled Cas9-sgRNA complex was assessed in a 30 μL reaction volume containing 30 nM of total sgRNA, 30 nM of Cas9 enzyme, and 3 nM of PCR-amplified DNA fragments containing the Cas9 target site. Agarose plugs were washed three times in 10 mM Tris–HCl (pH 8.0) and then incubated in 1 × Cas9 buffer for 2 h. For the MHC isolation, four agarose plugs (∼20 μg of genomic DNA) were used. Each plug was then divided into two equal volumes of 40 μL, and each was digested with one set of 60 μL pre-assembled Cas9-sgRNA mixes at 37 °C for 2 h. The reactions were terminated by replacing the reaction mixture with L buffer containing 0.5 mg/mL proteinase K and 1% (w/v) Sarkosyl, followed by incubation with gentle shaking at 4 °C for at least 1 h.

### Enrichment of the MHC sequence using pulsed-field gel electrophoresis

Following Cas9 digestion, the agarose plugs, along with H. wingei CHEF DNA Size Markers (Bio-Rad), were directly mounted onto the bottom edge of the gel comb and incorporated into 180 mL of 0.8% megabase resolution gel prepared in 1 × TAE buffer (Bio-Rad). Pulsed-field gel electrophoresis was carried out using a Bio-Rad CHEF-DR III system, operating at a 106° angle and 3 V/cm for 48 h, with a fixed switch time of 500 s. The gels were subsequently post-stained with 3 × GelGreen Stain in 0.1 M NaCl at room temperature for 30 min. Bands corresponding to approximately 2.3 Mb were excised from the gel and embedded into 4% low-melting-point agarose in 1 × TAE buffer. These samples were then subjected to a secondary pulsed-field gel electrophoresis under identical conditions (106° angle, 3 V/cm, fixed switch time of 500 s) for 17 h. Post-staining with 3 × GelGreen Stain in 0.1 M NaCl for 30 min at room temperature was repeated, and the high-molecular-weight DNA-containing bands were excised from the low-melting-point gels for subsequent recovery.

### Recovery of targeted high-molecular-weight DNA molecules

The recovery process for the targeted high-molecular-weight DNA molecules began with melting the recovered low-melting-point agarose gels. This was achieved by heating the gels in a heat block at 70 °C for 10 min, followed by an incubation at 42 °C for 5 min. Subsequently, 4 units of agarose (Thermo Fisher Scientific) were added to 100 mg (approximately 100 μL) of the molten 4% low-melting-point agarose. The mixture was gently combined and incubated at 42 °C for 45 min to digest the agarose, resulting in a high-molecular-weight DNA solution. Concurrently, 10 mL of TE buffer was prepared in a 6 cm Petri dish for each DNA sample. A 0.1 μm dialysis membrane (Millipore) was floated on the surface of the TE buffer for 15 min to hydrate the membrane. The high-molecular-weight DNA solution was then applied as a single drop onto the center of the hydrated dialysis membrane using a wide-bore tip and dialyzed at room temperature for 50 min. Following dialysis, the DNA was carefully transferred to a 1.5 mL tube using a wide-bore tip and quantified using the Qubit DNA high-sensitivity assay (Thermo Fisher Scientific). To verify the enrichment of the targeted region, quantitative real-time PCR was performed at multiple loci.

### Library construction and sequencing

Sequencing libraries for the 10 × Genomics platform were created using the Chromium Genome Library & Gel Bead Kit v2 (10 × Genomics) with specific modifications. To reduce barcode collisions, the targeted enriched high-molecular-weight DNA molecules were mixed 1:1 with the lambda DNA (NEB), and 200 pg of this mixed DNA was utilized for droplet generation. Subsequently, 30 μL of generated droplets were aliquoted for amplification and library construction following the manufacturer's instructions. This library was then sequenced on the Illumina HiSeq X Ten platform as per the manufacturer's guidelines.

HiFi SMRTbell library construction was carried out following the Ultra-Low DNA Input Workflow (PacBio) with some modifications. In brief, 20 ng of targeted, enriched, high-molecular-weight DNA was purified using magnetic beads (ProNex, Promega) and fragmented with g-Tubes (Covaris). The resulting library was size-selected to 8–12 kb using the BluePippin Size-Selection System, yielding a final library with an average size of 10 kb, which was then sequenced on the Revio System (PacBio).

### Preprocessing and alignment of sequencing data

The raw 10 × Genomics linked-read data were first processed with proc10xG (process_10xReads.py v0.0.2, regen_10xReads.py v0.0.1, lter_10x-Reads.py v0.0.1) (https://github.com/CeciliaDeng/proc10xG) to remove gem barcodes and then aligned to the lambda reference sequence using BWA (v.0.7.15) to eliminate unwanted lambda sequences. Unmapped reads were extracted for further filtration, removing those containing “N” bases or with fewer than three reads per barcode, and then converted back to their original format to restore barcode information. The processed data were then aligned to the GRCh38 reference using LongRanger (v.2.2.2), and phased variants were called with GATK (v.3.8).

For PacBio HiFi sequencing data, multiple sequencing runs for the same sample were merged using the pbbam toolkit (v.2.4.0) (https://github.com/PacificBiosciences/pbbam). Then, Pbmarkdup (v1.0.0) (https://github.com/PacificBiosciences/pbmarkdup) was used to remove duplicated reads from amplification. The PacBio HiFi reads were aligned to the GRCh38 reference using Minimap2 (v.2.26-r1175).^16^ Variant calling was performed with DeepVariant (v.0.10.0)^17^ and further re-genotyped using WhatsHap (v.2.3),^18^ and a consensus set of heterozygous variants was generated by intersecting those identified in both DeepVariant and WhatsHap.

### Haplotype-resolved assembly of the targeted MHC region

A reliable set of phased heterozygous variants was obtained by the intersection of variants identified in both 10 × Genomics linked-read and PacBio HiFi reads. Based on these phased variants, the PacBio HiFi reads from cell lines other than HeLa were separated into two haplotype-partitioned read sets using WhatsHap, along with untagged reads. To ensure uniformity in coverage, each haplotype-partitioned HiFi read set was downsampled by selecting the longest reads within each 10 kb window, aiming for a final coverage of 30 × per window.

Phased reads were then subjected to haplotype assembly using Hifiasm (v.0.19.7).^19^ Each haplotype-partitioned HiFi read set was merged with untagged reads to assemble the corresponding MHC haplotype. After preliminary assembly, contigs shorter than 50 kb were excluded. Due to the substantial polymorphism of the MHC region compared with the GRCh38 reference, certain HiFi reads from highly variable regions may have been omitted during the initial recruitment, resulting in potential assembly gaps. To address this, all HiFi reads were realigned to the preliminary assemblies to extract supplementary reads that were not initially aligned to the GRCh38 reference. These supplementary reads were subsequently combined with the originally recruited HiFi reads to construct the final haplotypes of the MHC region.

### Evaluation of haplotype-resolved assemblies of the MHC region

The assembled MHC haplotypes were aligned to the GRCh38 reference using Minimap2 to visualize the continuity across the targeted region. QUAST (v.5.2.0)^20^ was used to evaluate the assembly quality metrics, including total assembly length, length of the longest contiguous sequences, N50, NGA50, coverage, and duplication rates. The completeness of assembled MHC haplotypes was evaluated with BUSCO (v.5.7.0),^21^ which assessed evolutionarily conserved single-copy orthologs within the primates_odb10 database (v.2024-01-08). Additionally, Merqury (v.1.4.1)^22^ was employed to analyze the k-mer distribution between the assembled haplotypes and sequencing data. K-mer discrepancies refer to the differences between k-mer profiles of the original sequencing data and those derived from the assembled sequences, which can indicate errors, misassemblies, or incomplete regions in the assembly. To optimize the k-mer analysis, the k-mer value was calculated based on the length of the targeted region (4,548,949 bp) and determined to be 16.04. We then compared k-mer discrepancies between the sequences pre- and post-haplotype assemblies using this value.

### Characterization of genetic variants

Dipcall (v.0.3)^23^ was used to identify single nucleotide polymorphisms and short indels (insertions and/or deletions), with several parameters fine-tuned to optimize the process of variant calling: i) the xasm5 option was removed to ensure the inclusion of regions with high divergence; ii) the -z 200000, 1000 parameter was adjusted to enhance the contiguity of the alignment; and iii) -L 10000 was set to establish a minimum region length of 10 kb. The final variant data were exported in VCF format for downstream analysis. For the identification of chromosomal structural variants, we utilized SVIM-asm (v.1.0.3)^24^ with the diploid parameter.

### HLA and C4 typing

We used Immuannot (v.04/09/2024)^25^ for HLA and C4 typing. The assembled MHC haplotypes from each cell line were aligned using the immuannot.sh script against three built-in databases: IPD-IMGT/HLA (v.v3.44),^26^ IPD-KIR (v.2.13),^27^ and RefSeq (NG_011638.1).^28^ Multiple sequence alignments were performed and visualized using Mauve.^29^ When analyzing MHC class II sequences, the sensitivity of Mauve to the “seed weight” parameter was noted. A seed weight of 22 was selected to enable precise identification of reverse-complemented segments based on a previous study.^30^

### Generation of personal genome reference for each cell line

To create a personal genome reference for each cell line, we first removed the MHC region (chr6:28903952-33268517) from the GRCh38 reference. We then combined the remaining GRCh38 sequence with the assembled MHC haplotype sequences specific to each tumor cell line. The resulting personal genome reference for each cell line consisted of two reference sequences. Each sequence contained one of the cell-line-specific MHC haplotype sequences embedded within an otherwise intact GRCh38 reference.

### Evaluation of aneuploids of the MHC region

Quality control of whole-genome sequencing data was performed using Trim Galore (v.0.6.10) (https://github.com/FelixKrueger/TrimGalore) with the --paired parameter and default settings for base quality trimming (-q 20). This process removed adapter sequences and filtered out reads with base quality scores below 20. The quality-controlled data from each cell line was then aligned to cell-line-specific personal genome reference using the bwa mem command from BWA (v.0.7.15).^31^ After alignment, duplicated reads were removed using the MarkDuplicates module in GATK (v.4.2.0). Reads uniquely aligned to the haplotype-specific references (tagged with NM:i:0) were extracted using Samtools, while reads aligning to both haplotypes were filtered out.

To calculate sequencing depth, we used Samtools' depth function to determine the depth of uniquely aligned reads at each base position for each MHC haplotype. The dnadiff tool from MUMmer (v.3.0)^32^ was then used to compare and align the haplotype-specific sequence with the corresponding regions in the GRCh38 reference. This alignment allowed us to convert the calculated sequencing depth at each base position of each MHC haplotype to the GRCh38 coordinates, enabling visualization and cross-comparison of sequencing depth across the tumor cell lines. For each position with a genetic variant between the two haplotypes, we calculated the ratio of sequencing depth between the two haplotypes, used log2 to transform this ratio, and then smoothed it over a 10 kbp sliding window across the corresponding GRCh38 genome coordinates. To ensure continuity in the mapping process, we filled any gaps between consecutive regions by propagating the counts from the window endpoints.

## Results

### Targeted sequencing of the MHC region in five tumor cell lines

In this study, we selected five widely used tumor cell lines (A549, HeLa, HepG2, K562, and U2OS) based on their diverse lineages and extensive presence in scientific literature. i) A549, derived from basal epithelial cells of a 58-year-old Caucasian male with lung adenocarcinoma,^33^ serves as a stable *in vitro* model for human alveolar type II pulmonary epithelium.^34^ ii) HeLa, the first immortal human cell line, was isolated in 1951 from a cervical carcinoma of a 31-year-old patient.^35^ Of epithelial origin, HeLa is renowned for its robust growth and has been utilized in over 57,000 publications for both normal and oncological research.^36^, ^37^, ^38^ iii) HepG2, a hepatoblastoma cell line derived from a 15-year-old European male, represents the human endodermal lineage.^39^^,^^40^ It has contributed over 2000 datasets to the Encyclopedia of DNA Elements Project (ENCODE).^41^ iv) K562, established in 1970 from a 53-year-old Caucasian female with chronic myelogenous leukemia, was the first human immortalized myelogenous leukemia cell line.^42^ v) U2OS, an osteosarcoma cell line with epithelial morphology, was cultivated in 1964 from the tibia of a 15-year-old Caucasian female.^43^

We applied a previously developed strategy for haplotype-resolved assembly of the targeted MHC regions^14^ to these five tumor cell lines. In brief, CRISPR-based in-gel digestion was utilized for targeted enrichment of the 4.3 Mb MHC region (chr6: 28903952–33268517) (Fig. 1A; Fig. S1A). The excision was guided by sgRNAs designed to target the non-polymorphic DNA sequences flanking the MHC region (Fig. 1B; Fig. S1B). The efficiency of targeted enrichment among various cell lines was evaluated using quantitative real-time PCR on genomic loci located within or adjacent to the MHC region. All three loci within the targeted region (*HLA-E*, *TCF-19*, *MICB*) exhibited more than fivefold enrichment, while no enrichment was observed in the MHC-flanking locus (*RNF8*) (Fig. 1C).

Enriched high-molecular-weight DNA molecules of each cell line were subjected to 10 × Genomics linked-reads and PacBio CCS HiFi sequencing. Sequencing reads were aligned to the GRCh38 human reference genome, and the efficiency of targeted enrichment was further evaluated by calculating the coverage depth of sequencing reads. Consistent with the quantitative real-time PCR results, A549 and U2OS cells exhibited the highest enrichment among the five investigated cell lines, while HepG2 cells showed the lowest enrichment (Fig. 1D; Fig. S2).

### Haplotype-resolved assembly of targeted MHC region in cell lines

We assembled the MHC haplotypes of each cell line by integrating 10 × Genomics linked-reads with PacBio CCS HiFi sequencing data^14^ (Fig. 1A). Initially, linked-reads and PacBio HiFi reads were aligned separately to the GRCh38 reference genome to identify the heterozygous variants. A highly reliable set of phased heterozygous variants was obtained by the intersection of variants identified in both sequencing platforms (Table S1). We noticed that the number of heterozygous variants identified in the HeLa cell line is much smaller compared with the number of variants identified in the other four cell lines (Table S1). This is consistent with previous reports that the HeLa cell line exhibits a loss of heterozygosity in the MHC region on the short arm of chromosome 6^8^, while the other four cell lines retain the short arm of chromosome 6 from both parents based on karyotyping.^8^^,^^9^^,^^44^, ^45^, ^46^ Therefore, in the following assembly process, we treated the HeLa cell line as haploid and the other cell lines as diploid.

**Figure 2:**
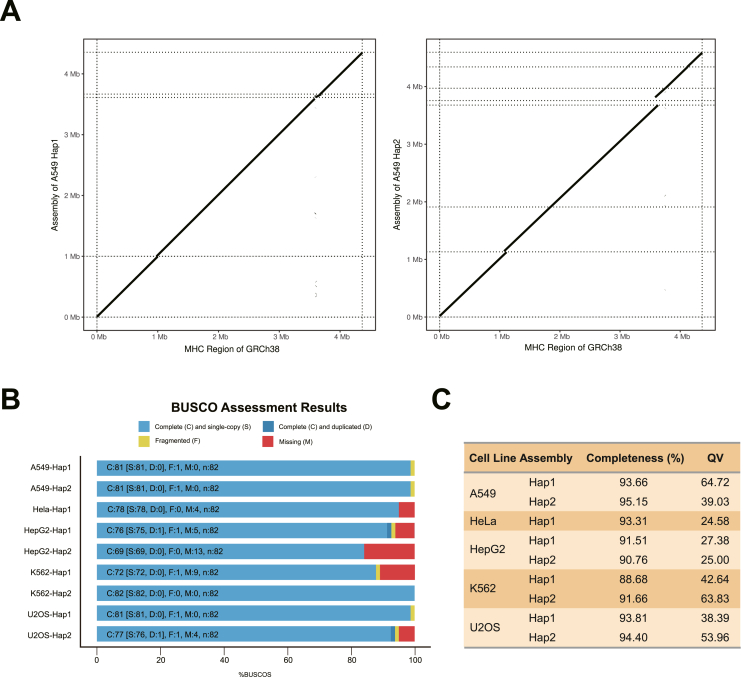
Assessment of the haplotype-resolved MHC assemblies of the tumor cell lines. **(A)** The continuity of targeted assembled MHC haplotypes of the A549 cell line by comparing with the MHC region of the GRCh38 reference. The Y-axis illustrates the coordinates from the targeted assembly, whereas the X-axis denotes the coordinates from the GRCh38 reference. **(B)** The completeness of assembled MHC haplotypes for each cell line evaluated with BUSCO. The bar chart illustrates the proportions of the various states of the CEGs within the assemblies. “n” represents the total count of CEGs within the MHC region. **(C)** The Merqury evaluation of the completeness of assembled MHC haplotypes. Quality value (QV) was determined by a log-scaled probability of error for the consensus accuracy.

For the four diploid cell lines (A549, HepG2, K562, and U2OS), the phased heterozygous variants were used to separate the HiFi reads into two haplotype-partitioned read sets. These read sets, together with untagged HiFi reads, were then used to assemble the two MHC haplotypes for each cell line. To further refine the assembly, all HiFi reads were realigned to the preliminary assembled sequences, allowing for the extraction of supplementary HiFi reads for a secondary round of assembly. Eventually, the MHC haplotypes of five cell lines were assembled separately, with lengths of each haplotype ranging from 4.1 Mb to 4.7 Mb, approximating the targeted length of 4.3 Mb based on the GRCh38 reference (Table 1). We compared each assembled haplotype of the cell lines to the GRCh38 reference and observed good contiguity across most of the targeted region (Fig. 2A; Fig. S3), with the number of contigs varying between 5 and 10 for each haplotype (Table 1).

**Table 1:** Statistics of assembled MHC haplotypes in tumor cell lines.

Cell line	Haplotype	Contigs	Largest contig length (bp)	Total length (bp)	Coverage (%)	NGA50	LGA50	Duplication ratio (%)
A549	Hap1	6	2611266	4502994	95.859	1107893	2	1.02
	Hap2	8	1768330	4650717	96.316	759751	3	1.023
U2OS	Hap1	9	2460957	4496367	97.44	456503	3	1.008
	Hap2	9	1972207	4513815	96.33	412383	4	1.007
HepG2	Hap1	9	2090983	4282982	95.835	431846	3	1.003
	Hap2	16	1668242	4088651	92.048	342821	4	1.001
K562	Hap1	10	861931	4329141	92.586	422928	4	1.006
	Hap2	5	2531885	4305725	96.372	581447	3	1.001
Hela	Hap1	6	3306066	4491294	98.031	485095	4	1.01

### Evaluation of haplotype-resolved assemblies of the MHC region across five cell lines

We conducted multiple assessments to evaluate the assembled MHC haplotypes of the five cell lines. Assembly metrics results from QUAST^20^ (Table 1) shows that all the assembled haplotypes not only cover most of the MHC region in the GRCh38 reference genome but also achieve an NGA50 exceeding 0.3 Mbp, where NGA50 represents the length where the set of aligned contigs of this length or longer covers at least 50% of the reference genome, and serves as a widely used indicator of assembly contiguity and accuracy, with higher values reflecting better quality. Additionally, the duplication ratios are close to 1, indicating little erroneously duplicated content in the assemblies. Among assembled MHC haplotypes, the A549 cell line has the best contiguity, with its MHC haplotigs achieving NGA50 lengths of 1.1 Mbp and 0.76 Mbp, respectively, while the MHC haplotypes of the HepG2 cell line show poorer assembly quality, which may be the result of relatively low sequencing coverage.

We employed BUSCO^47^ to evaluate the completeness of the assembled MHC haplotypes. Orthologous core eukaryotic genes (CEGs) are highly evolutionarily conserved and present in low copy numbers in higher eukaryotes, making them a critical metric for evaluating the quality of assemblies.^48^ The completeness of 82 CEGs in the targeted MHC region indicated that the assembled MHC haplotypes of the five cell lines achieved approximately 90% completeness (Fig. 2B). Specifically, both MHC haplotypes of the A549 cell line reached a completeness of 98.8%, each with only one CEG appearing fragmented. In contrast, haplotype 1 of the HepG2 cell line showed lower completeness, with 13 out of the 82 CEGs missing. Considering that BUSCO's performance is based on only 82 CEGs, which may not be sufficient to evaluate sub-chromosomal regional assemblies, we further conducted another reference-free assessment, Merqury,^22^ which analyzes k-mers (short, fixed-length nucleotide sequences that serve as unique genomic identifiers). Using haplotype-specific k-mers, which represent sequences unique to each haplotype, our evaluation revealed that most of the assembled MHC haplotypes achieved at least 90% completeness (Fig. 2C). Among these, the assembled MHC haplotypes of the A549 cell line are notably better, with completeness rates of 93.7% and 95.2% in covering the original reads' k-mers. Additionally, the A549 cell line exhibits the lowest haplotype switch errors, as indicated by quality values.

We next performed HLA typing on the assembled MHC haplotypes using Immuannot.^25^ For the three major HLA class I genes, all but one reached the 8-digit resolution with zero edit distance (Table 2). These results are consistent with the 4-digit typing results inferred from RNA-seq data,^49^^,^^50^ but with more complete and higher resolution. In the haplotype 2 of the A549 cell line, we observed the presence of the rare *HLA-Y* gene, which is absent in the GRCh38 reference genome due to its common gene deletion,^51^ along with *HLA-A*∗30 (Table S2). This is consistent with a previous report showing that *HLA-Y* is highly associated with *HLA-A*∗29, ∗30, ∗31, ∗33, or ∗68.^25^ In the HLA class II region, we resolved the DR haplotype structures based on the combination of *HLA-DRB1* and *DRB3/4/5* genes.^52^ The A549, K562, and HepG2 cell lines contain DR3 haplotype (*DRB1*∗11, ∗03, ∗14 with *DRB3*). The structural homology of the assembled *DRB3*-containing haplotypes was confirmed through multiple sequence alignments (Fig. S4). Additionally, the K562 cell line also harbors the DR4 haplotype (*DRB1*∗04 with *DRB4*), while the HepG2 cell line carries the DR2 haplotype (*DRB1*∗15 with *DRB5*). In contrast, the HeLa cell line was observed to possess the DR1 haplotype (*DRB1*∗01) without any associated *DRB3/4/5*. Notably, the upstream and downstream regions of *DRB5* in the DR2 haplotype of the HepG2 cell line exhibit high homology with the corresponding regions in the DR1 haplotype of the HeLa cell line, which may reflect their common evolutionary origins and functional similarities. We also determined the *C4* genotypes (Table S3), which are part of the complement system.^53^^,^^54^ These *C4A* and *C4B* alleles are arranged in the configuration of *C4A-L* ∼ *C4B-L* ∼ *C4A-S* ∼ *C4B-S*, similar to previous observations.^55^

**Table 2:** HLA typing of three classical HLA class I genes using assembled MHC haplotypes.

Cell line	Haplotype	HLA-A	Edit distance	HLA-B	Edit distance	HLA-C	Edit distance
A549	Hap1	25:01:01:01	0	18:01:01:05	0	12:03:01:01	0
	Hap2	30:01:01:01	0	44:03:01:01	0	16:01:01:01	0
K562	Hap1	31:01:02:01	0	40:01:02:04	0	03:04:01:01	0
	Hap2	11:01:01:01	0	18:01:01:01	0	05:01:01:01	0
HepG2	Hap1	02:01:01:01	0	51:08:01:01	0	16:02:01:01	0
	Hap2	24:02:01:01	0	35:14:01	0	04:01:01:79	0
U2OS	Hap1	02:01:01:01	0	44:27:01:01	0	07:04:01:01	0
	Hap2	32:01:01:01	0	44:02:01:01	0	05:01:01:02	0
Hela	Hap1	68:02:01:01	0	15:03:01:02	0	12:03:01:02	0

### Characterization of genetic variants using haplotype-resolved assemblies

**Figure 3:**
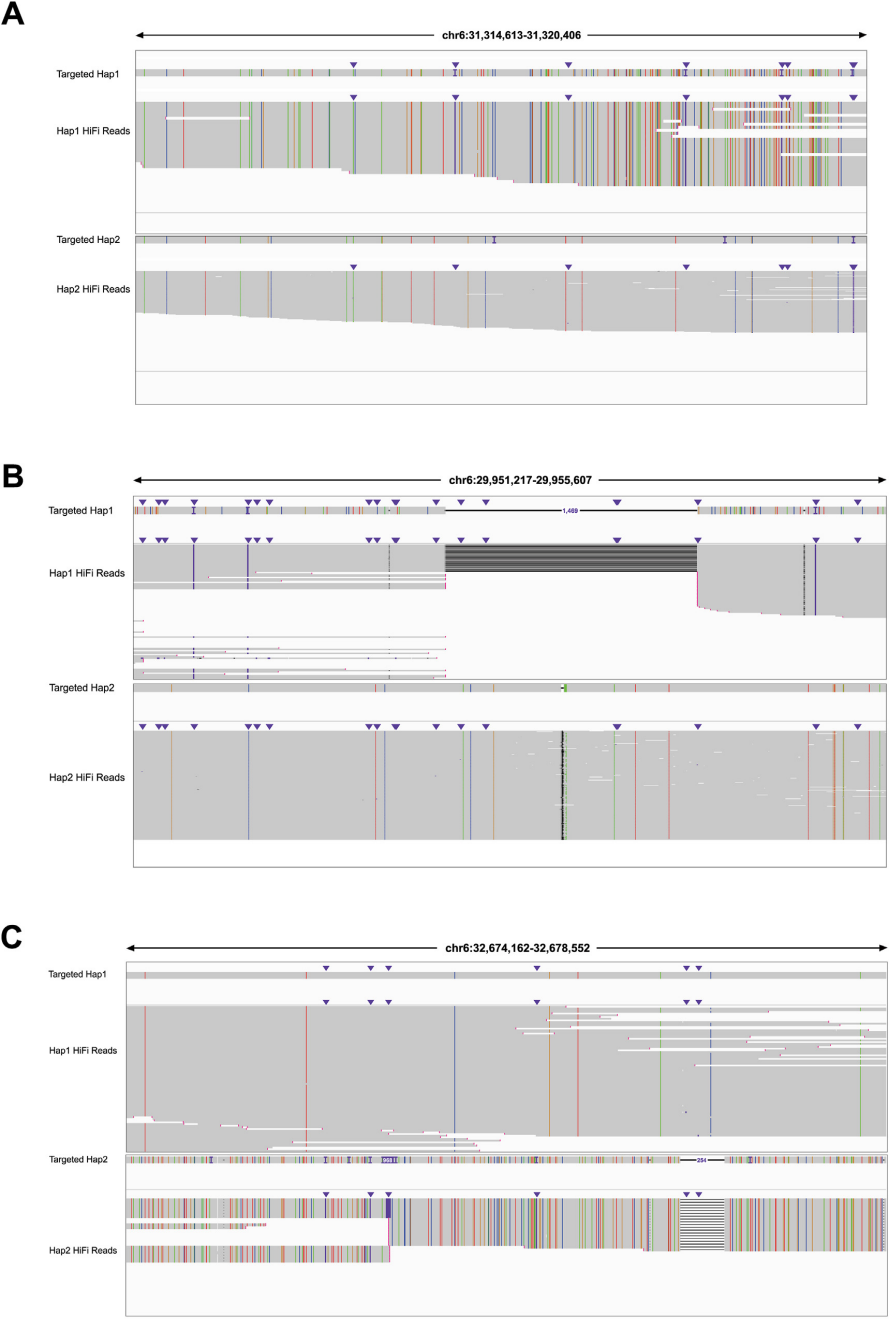
Genetic variants identified using the haplotype MHC assemblies in the K562 cell line. **(A)** An example of a highly polymorphic region with identified variants in the assembled haplotypes of the K562 cell line. Displayed below are high-confidence HiFi reads with identified variants. Colorful lines and dots denote the four types of nucleotides that are distinct from those in the GRCh38 reference. Specifically, purple “I” characters and dots indicate small insertions (<50 bp), while black dots mark small deletions (<50 bp). **(B)** A 1469 bp deletion on haplotype 1 of K562, supported by HiFi reads. **(C)** A 968 bp insertion on haplotype 2 of K562, supported by HiFi reads.

With haplotype-resolved assemblies of the MHC region available, we can now characterize genetic variants of the MHC region in these five cell lines more accurately and comprehensively.^56^ We identified a large number of single nucleotide polymorphisms and indels across the five cell lines (Table S4). Many of them are located in highly polymorphic or repetitive regions, and their accuracies are supported by high-confidence HiFi reads (Fig. 3A; Fig. S5). In all five cell lines, the most polymorphic parts of the MHC locus are located in regions surrounding three HLA class I genes (*HLA-A*, *HLA-B*, and *HLA-C*) and three HLA class II genes (*HLA-DR*, *HLA-DQ*, and *HLA-DP*) (Fig. S6). Assembled MHC haplotypes also facilitate the detection of large structural variants (Table S5), which are difficult to determine using short-read sequencing. For instance, we identified a 1469 bp deletion in the region around 29.95 Mb on haplotype 1 of K562 (Fig. 3B) and a 968 bp insertion in the region around 32.67 Mb on haplotype 2 of K562 (Fig. 3C), both of which are supported by the presence of HiFi long reads. Unlike with single nucleotide polymorphisms and indels, we identified more structural variants in the *HLA-DR* and *HLA-DQ* regions compared with the HLA class I region across all five cell lines (Fig. S7).

### Aneuploidy of the MHC region revealed through assembled haplotypes of cell lines

**Figure 4:**
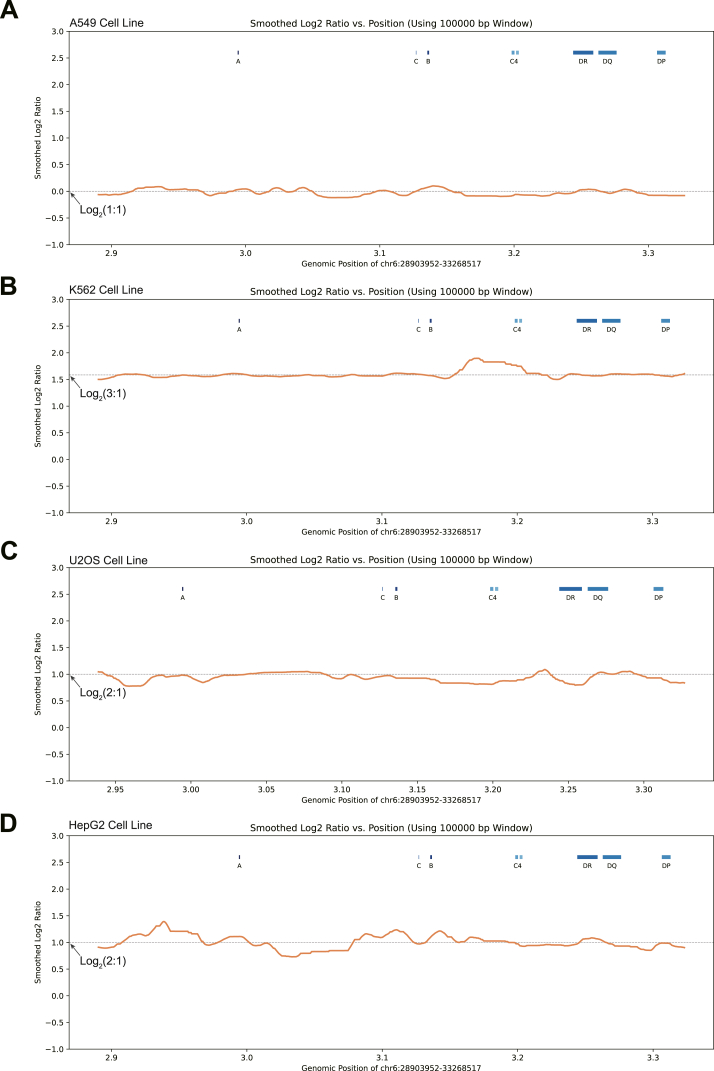
Aneuploidy of the MHC region revealed through assembled haplotypes of cell lines. **(A**–**D)** Aneuploidy in the A549 (A), K562 (B), U2OS (C), and HepG2 (D) cell lines were visualized using the log2 transformed sequencing depth ratio between the two assembled MHC haplotypes. Orange lines represent the results smoothed over a 10 kb window. Dotted lines indicate the overall ratios between two haplotypes, with the values shown on the left. Blue boxes represent regions corresponding to three HLA class I genes (*HLA-A*, *HLA-B*, and *HLA-C*), *C4* genes, and three HLA class II genes (*HLA-DR*, *HLA-DQ*, and *HLA-DP*).

Tumor cells are commonly characterized by changes in the copy number of entire chromosome arms, a phenomenon termed aneuploidy.^57^^,^^58^ To characterize aneuploidy of the MHC region in these tumor cell lines, we first generated a personal genome reference for each cell line by replacing the MHC sequence in the GRCh38 reference with the corresponding MHC haplotype sequences (Fig. S8). We aligned whole-genome sequencing data of each cell line^59^^,^^60^ separately to the GRCh38 reference genome and the cell-line-specific personal genome references. The total number of mapped reads across the entire genome was nearly identical between these references (<0.01% difference), consistent with previous observations of minimal overall mapping differences when haplotype-specific MHC references are used.^30^ However, when examining the MHC region specifically, we observed a notable increase in the number of sequencing reads that aligned to the personal genome references, showing improvements of 2.32%–3.03% compared with GRCh38 (Table S6). This suggests that while the impact on overall whole-genome alignment is modest, the use of personal genome references with haplotype-specific MHC assemblies enhances the resolution of MHC-specific reads. We retained reads uniquely aligned to the haplotype-specific MHC references for subsequent sequencing depth calculation of the MHC region. In the A549 cell line, we observed a 1:1 ratio of the sequencing depth between haplotype 1 and haplotype 2 (Fig. 4A). In contrast, we observed an overall 2:1 ratio in both HepG2 and U2OS cell lines (Fig. 4C, D). These findings are consistent with previous karyotype results using GTG-banding-based optical imaging techniques.^45^ However, we observed a 3:1 ratio between the two haplotypes in the K562 cell line (Fig. 4B), differing from the 2:1 ratio observed in most existing karyotype results.^9^ This inconsistency may stem from a disputed chromosomal fragment potentially originating from chromosome 6.^61^ These aneuploidy results further underscore the reliability of our assembled MHC haplotypes in these tumor cell lines.

## Discussion

In this study, we constructed haplotype-resolved assemblies of the highly polymorphic and structurally diverse MHC region in five widely used tumor cell lines (A549, HeLa, HepG2, K562, and U2OS). The assembled MHC haplotypes range from 4.1 Mb to 4.7 Mb in length and demonstrate good continuity when compared with the GRCh38 reference genome. An evaluation of the assembly quality using tools such as QUAST, BUSCO, and Merqury showed that the assembled MHC haplotypes in the five cell lines achieved approximately 90% completeness, with the A549 cell line exhibiting the highest assembly quality. Comprehensive analyses of genetic variants identified a large number of single nucleotide polymorphisms, indels, and structural variants, with most located in regions surrounding major HLA class I and II genes, highlighting the pronounced polymorphisms of these gene regions.^62^ Finally, using the assembled MHC haplotypes as references, we examined the aneuploidy of the MHC region in these cell lines.

Our study lays a robust groundwork for future research and offers several immediate implications. These five cell lines are widely employed as models in studies of molecular mechanisms, large-scale genetic screenings, and evaluations of anti-tumor therapies and have generated thousands of multi-omics datasets. High-quality assembled MHC haplotypes could be used to refine expression results in cell lines, reducing alignment biases caused by reference mismatches and uncovering haplotype-specific functional effects.^63^ Incorporating our assemblies into genomic resources such as IMGT/HLA or tools could improve the accuracy of transcriptomic analyses by enabling alignment to specific MHC haplotypes rather than a single reference genome. With the availability of high-quality assembled MHC haplotypes, researchers can now reanalyze these data to further elucidate the roles of the MHC region in various immune processes. Moreover, the haplotype-resolved MHC assemblies from these cell lines enable investigations into allele-specific epigenetic regulation and expression of the MHC region across different cellular states. Haplotype-resolved MHC assemblies may offer valuable insights into how specific alleles influence chromatin accessibility, DNA methylation, and histone modifications, epigenetic factors that govern gene expression. By integrating these assemblies with epigenomic data (*e.g.*, ATAC-seq or ChIP-seq), researchers can delineate the regulatory landscapes of critical MHC genes like *HLA-DRB* and *HLA-DQ*. Linking haplotype-specific expression to epigenetic features may also uncover novel mechanisms of immune cell differentiation and activation. For instance, prior studies have noted significant differences in MHC gene expression among individuals carrying autoimmune disease-associated alleles, such as the *HLA-DRB1* shared epitope, compared with those without.^64^ These findings suggest that both structural variations and epigenetic regulation contribute to allelic effects in autoimmune diseases. Furthermore, cell-line-specific haplotype assemblies provide a valuable model for examining how MHC gene regulation shifts under various conditions, including immune activation, cytokine signaling, and exposure to pathogens. This enables researchers to explore allele-specific responses *in vitro*, linking functional genomics to clinical insights into MHC-linked disease susceptibility. When incorporated into publicly available genomic databases, these resources become powerful tools for investigating the interplay between genetic variation, epigenetic regulation, and immune function. This advancement will contribute to a more comprehensive understanding of MHC gene function and regulatory mechanisms, as well as their specific roles in immune responses.^65^

Despite our findings, there are still some technical limitations. Firstly, variations in sequencing depth among different cell lines may affect the continuity and completeness of assembled MHC haplotypes. For example, we observed relatively low enrichment for the MHC regions of HepG2 and K562 cell lines (Fig. S2), which may account for the relatively poor assembly quality in these cells. This depth variation may result from differences in sample processing, and increasing sequencing depth or integrating additional strand-specific sequencing technologies could be beneficial.^66^ It is worth noting that we observed a notable reduction in coverage for the MHC class II region (positions 33.2–33.5 Mb; Fig. 1D; Fig. S2), likely due to high polymorphism and potential structural variants that diverge from the GRCh38 reference genome.^67^^,^^68^ Since the MHC class II region is more polymorphic than MHC class I, fewer reads map accurately, resulting in lower coverage. However, when we aligned HiFi reads to our cell-line-specific MHC haplotypes, coverage in this region became more uniform and increased substantially (Fig. S9), suggesting that reference mismatch is the primary cause of the observed coverage reduction. Secondly, although our targeted enrichment and assembly approach performs well in handling most of the MHC region, there is still room for improvement in the assembly strategy for highly polymorphic regions, such as the MHC class II region. The recruitment of HiFi reads aligned to the MHC region of the GRCh38 reference may lead to the omission of reads belonging to these highly polymorphic regions, resulting in gaps and fragmentation in the assemblies.

## CRediT authorship contribution statement

**Haozhe Yuan:** Writing – original draft, Formal analysis, Data curation. **Mengping Jiang:** Data curation. **Xingyu Xu:** Methodology, Formal analysis. **Jialiang Zhu:** Methodology. **Shulong Dong:** Methodology. **Weida Meng:** Methodology. **Dandan Zhang:** Formal analysis. **Jiakang Ma:** Data curation. **Yicheng Lin:** Formal analysis. **Ziqiang Chen:** Formal analysis. **Shaoyang Sun:** Data curation. **Wenqing Qiu:** Methodology. **Yun Liu:** Writing – review & editing, Supervision, Project administration, Investigation, Funding acquisition, Conceptualization.

## Data availability

10 × Genomics linked-read data (accession code no. SRR31728049, SRR31728050, SRR31728051, SRR31728052, SRR31728053) and the PacBio HiFi read data (accession code no. SRR31728044, SRR31728045, SRR31728046, SRR31728047, SRR31728048) were deposited into the Sequence Read Archive (SRA). Whole-genome sequencing reads of the cell lines were acquired from the Encyclopedia of DNA Elements (ENCODE) database (A549 cell line: ENCSR521ELB; HepG2 cell line: ENCSR319QHO; K562 cell line: ENCSR711UNY)^59^ and Gene Expression Omnibus (GEO) database (U2OS cell line: SRX2357008).^60^

## Code availability

The assembled MHC haplotypes and all the codes used to assemble and evaluate the MHC haplotypes are available at https://github.com/Haozhe-Yuan/MHC_Haplotype_Assemble.

## Funding

This work was supported by funding from the 10.13039/501100003399Science and Technology Commission of Shanghai Municipality, China (No. 23JS1400400), the 10.13039/501100001809National Natural Science Foundation of China (No. 32300484, 82171837), Shanghai Municipal Science and Technology Major Project (China) (No. 2017SHZDZX01, 2018SHZDZX01), and ZJLab (Shanghai, China).

## Conflict of interests

The authors declared no conflict of interests.
