# Supplementary material for: Haplotype-resolved assemblies of the MHC region in five widely used tumor cell lines
Source: Genes Dis. 2025 Mar 18;12(5):101603. doi: 10.1016/j.gendis.2025.101603 (PMC12212132; doi:10.1016/j.gendis.2025.101603)
Supplement: Multimedia component 1 [file mmc1.pdf]

Supplementary Figures

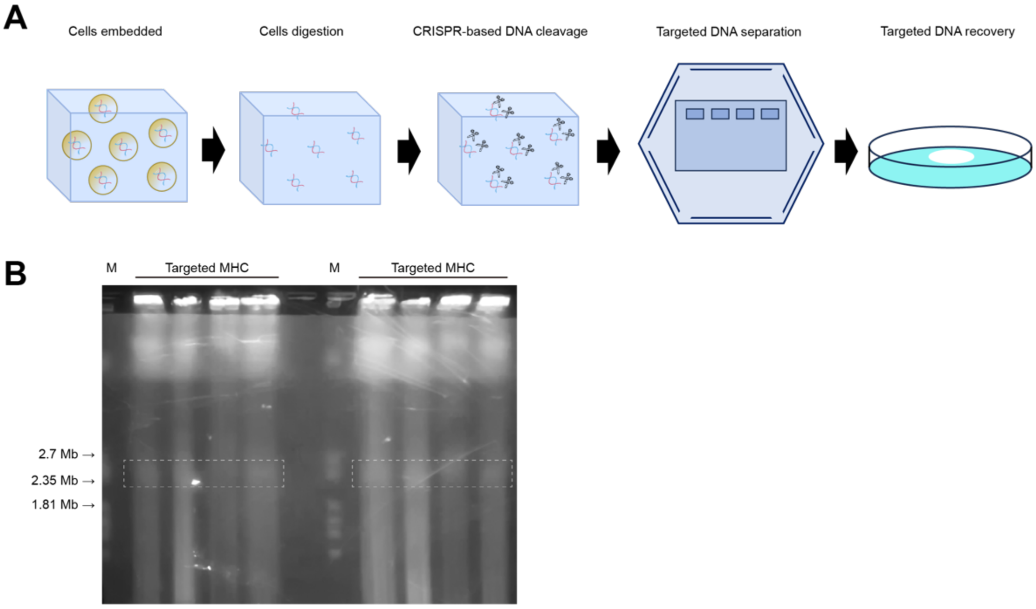

Figure S1 The CRISPR-based targeted enrichment of the MHC region of the tumor cell lines

(A) Schematics of the CRISPR-based targeted enrichment. Cells were embedded in agarose plugs and subjected to proteinase K digestion. The specific genomic region of interest was cleaved by CRISPR-mediated in-gel digestion, followed by separation using pulsed-field gel electrophoresis (PFGE). The high-molecular-weight (HMW) MHC molecules were subsequently recovered by dialysis. **(B)** The DNA molecules from the MHC region were cleaved using CRISPR-based in-gel digestion and subsequently separated by PFGE. Gel bands approximately ~2.3 Mb in size, identified with H. wingei CHEF DNA Size Markers (M) and highlighted by gray dashed boxes, were excised to isolate high-molecular-weight (HMW) MHC molecules.

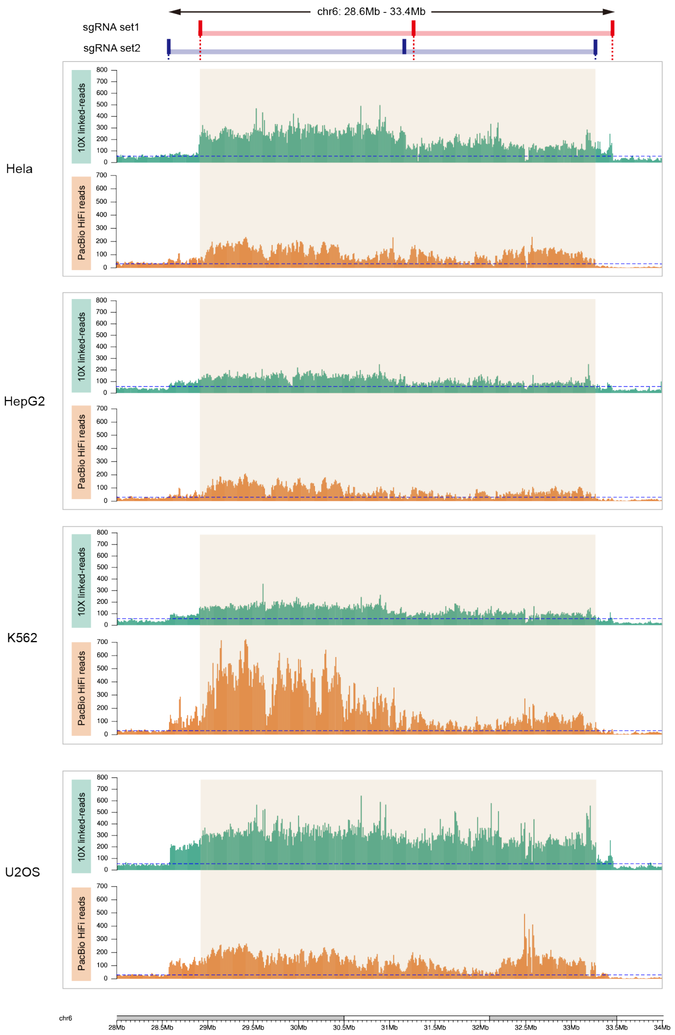

Figure S2 The sequencing coverage of the targeted MHC region

The overall coverage of sequencing reads mapped to the GRCh38 reference for the targeted MHC region in the HeLa, HepG2, K562, and U2OS cell lines, obtained from 10x Genomics and PacBio HiFi sequencing platforms. The beige area, encompassed by the two sets of sgRNAs (red and blue bars at the top), highlights the targeted MHC region. The dashed lines indicate a 50-fold enrichment for 10x Genomics linked-read data (green) and a 30-fold enrichment for PacBio HiFi data (orange).

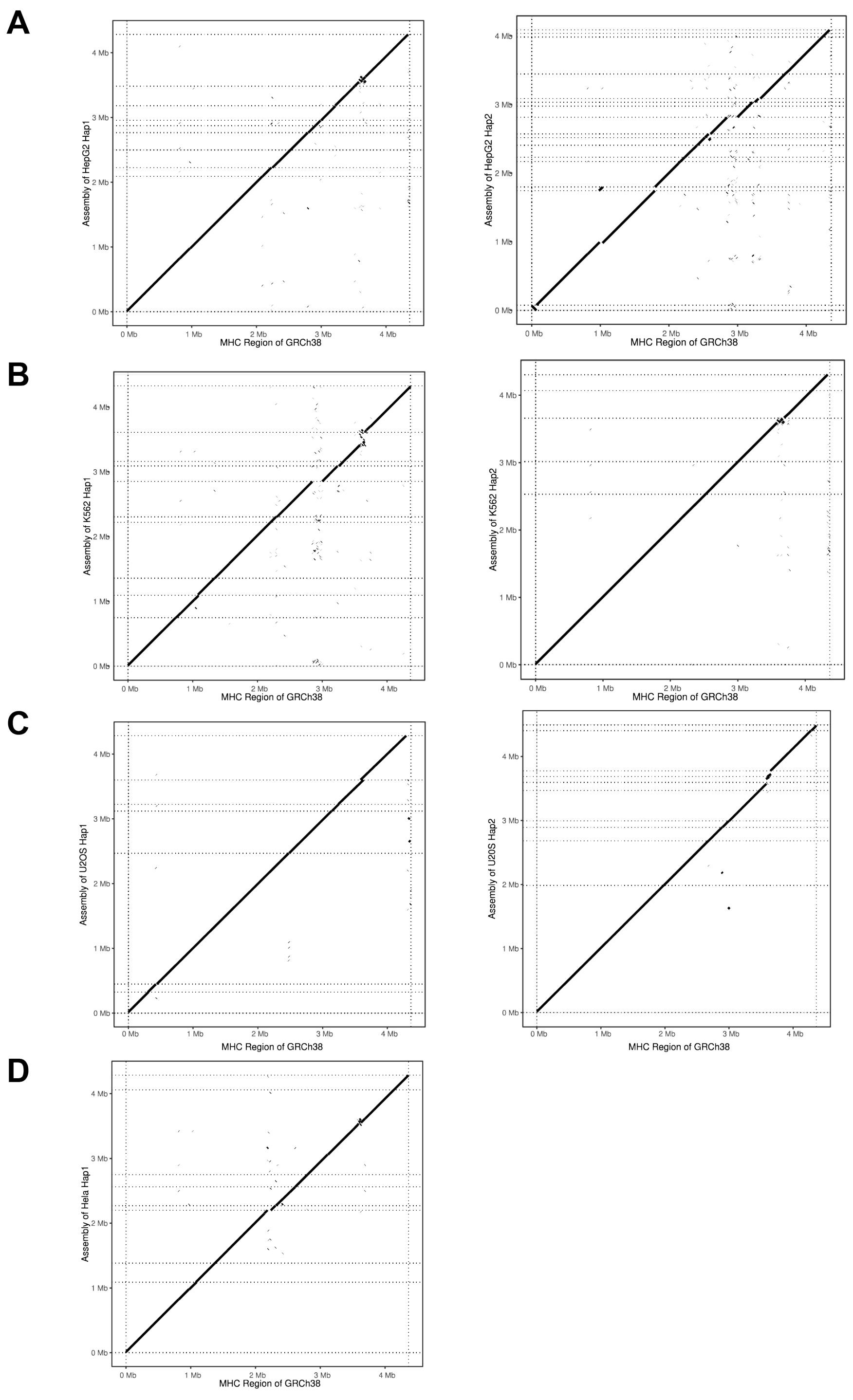

Figure S3 The continuity of targeted assembled MHC haplotypes of the tumor cell lines.

(A) HepG2, **(B)** K562, **(C)** U2OS, and **(D)** Hela cell lines. The Y-axis represents the coordinates from our targeted assembly, while the X-axis corresponds to the coordinates of the GRCh38 reference genome.

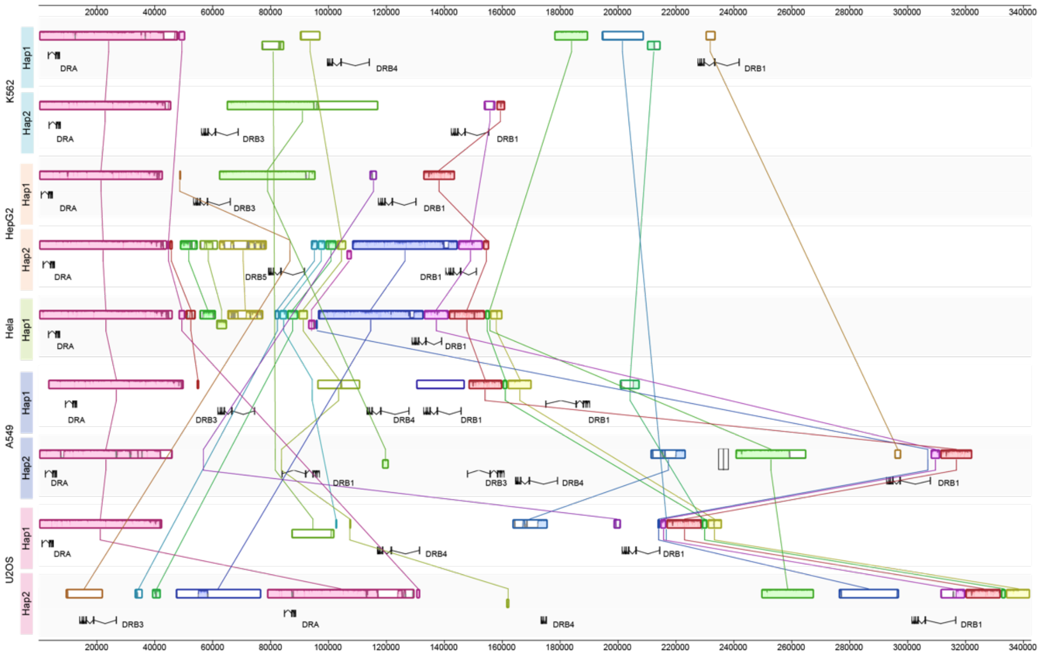

Figure S4 Visualization of MHC II Haplotype Structures.

Visualization of multiple-sequence alignments of MHC II haplotype structures from the five tumor cell lines. Colors indicate sequence similarity across haplotypes, while segments beneath the respective plots denote inversions. The visualization was generated using Mauve with the "seed weight" parameter set to 22. For clarity, vertical lines linking horizontally aligned homologous regions were refined.

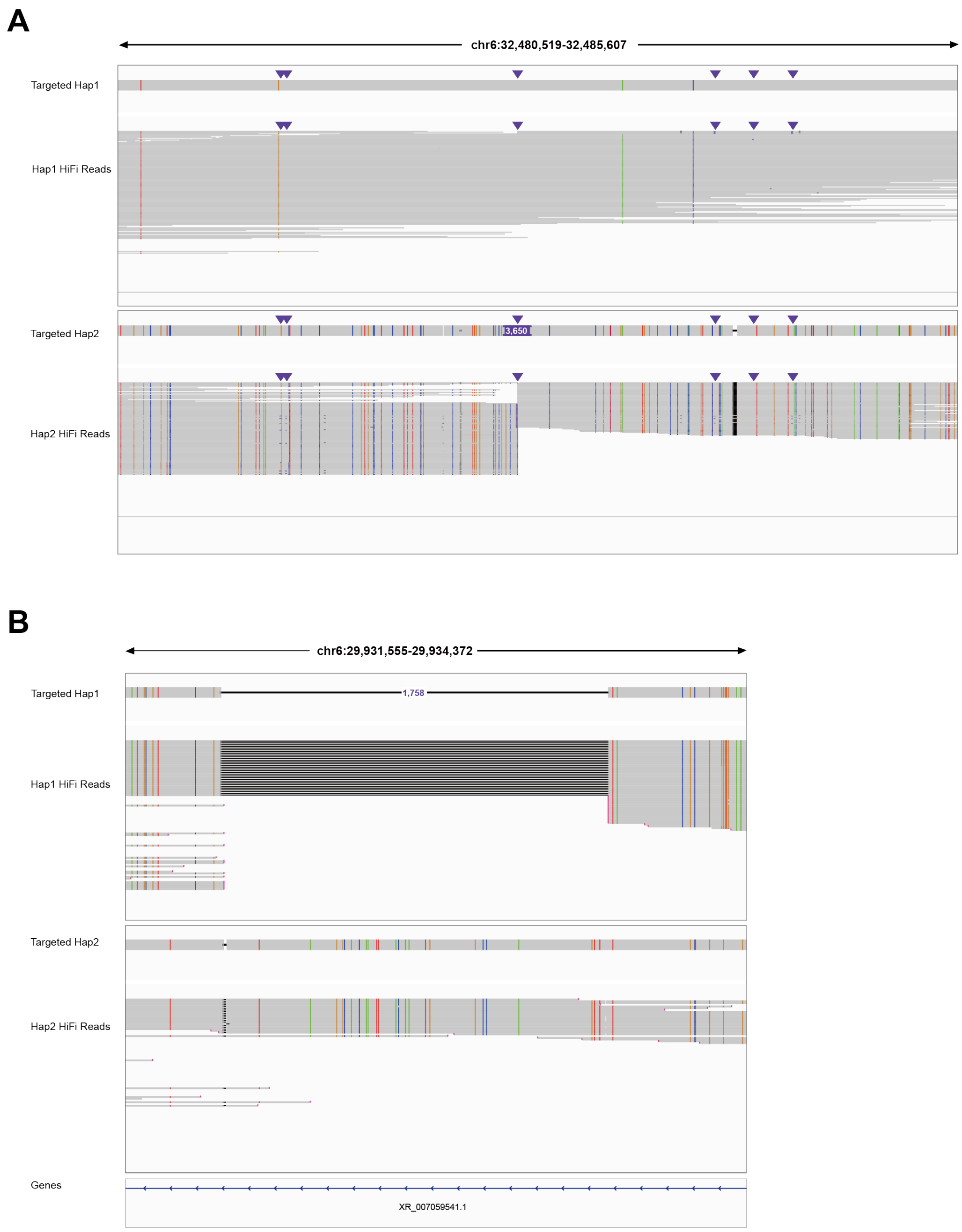

Figure S5 Genetic variants identified using the haplotype MHC assemblies in the A549 cell line

(A) A 3,650 bp insertion on haplotype 2 of the A549 cell line, supported by HiFi reads.

(B) A 1,758 bp deletion on haplotype 1 of the A549 cell line, supported by HiFi reads.

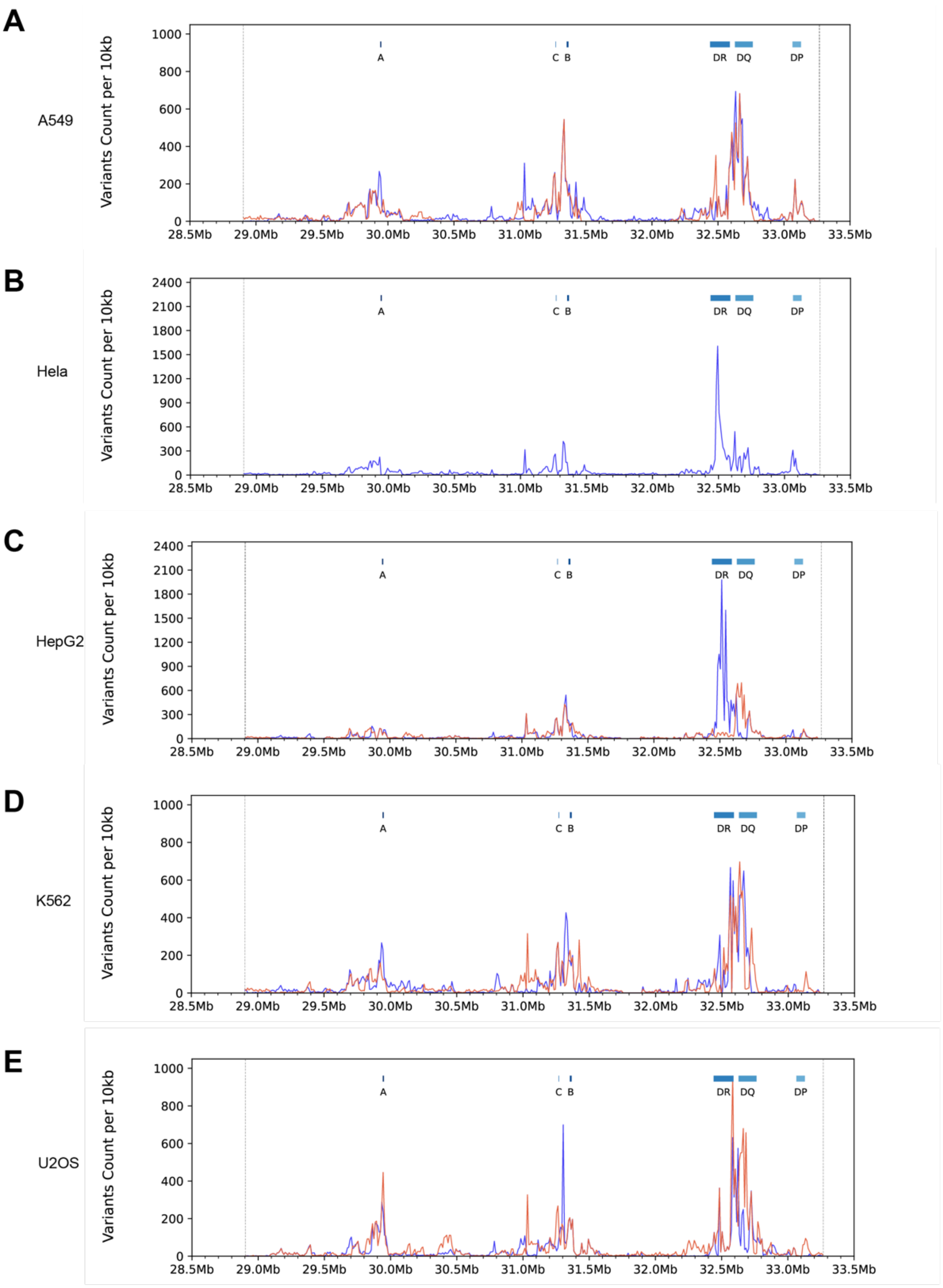

Figure S6 The density plots of genetic variants throughout the targeted MHC region in tumor cell lines.

The X-axis indicates the coordinates of the targeted MHC assemblies of the A549 **(A)**, Hela **(B)**, HepG2 **(C)**, K562 **(D)** and U2OS **(E)** cell lines, and the Y-axis indicates the

number of genetic variants (SNPs and InDels) relative to hg38 reference in each 10 kb window. Blue line: the assembly of haplotype 1; red line: the assembly of haplotype 2.

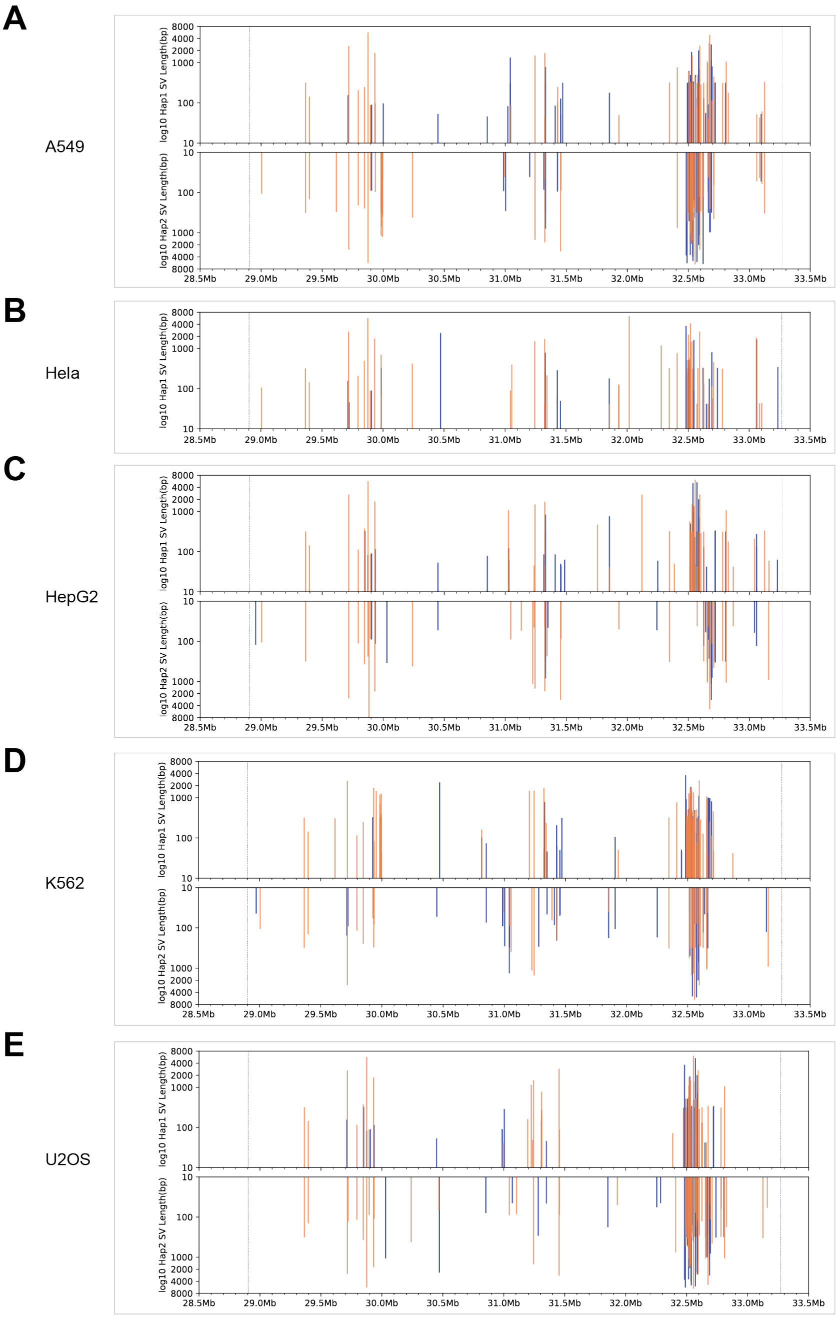

Figure S7 The distribution of structural variants (SVs) in tumor cell lines.

Distribution of haplotype-specific SVs in the A549 **(A)**, Hela **(B)**, HepG2 **(C)**, K562 **(D)** and U2OS **(E)** cell lines. The Y-axis shows the log10-transformed lengths of the structural variants. The upper half of each subplot displays the distribution of SVs for haplotype 1, while the lower half shows the distribution for haplotype 2. Blue lines: insertion variants; orange lines: deletion variants.

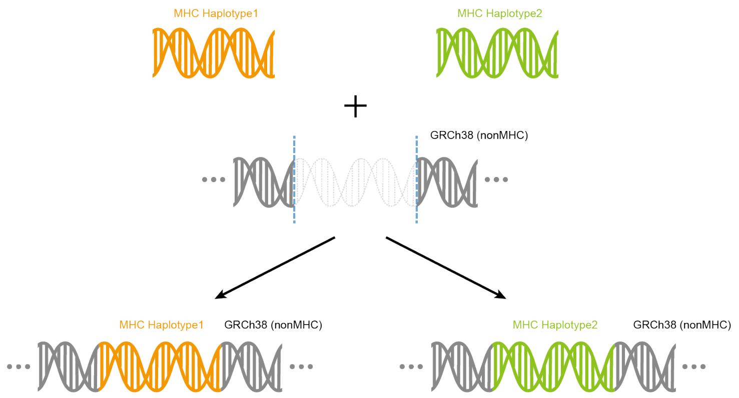

Figure S8 Schematics of generating personal genome reference for each tumor cell line.

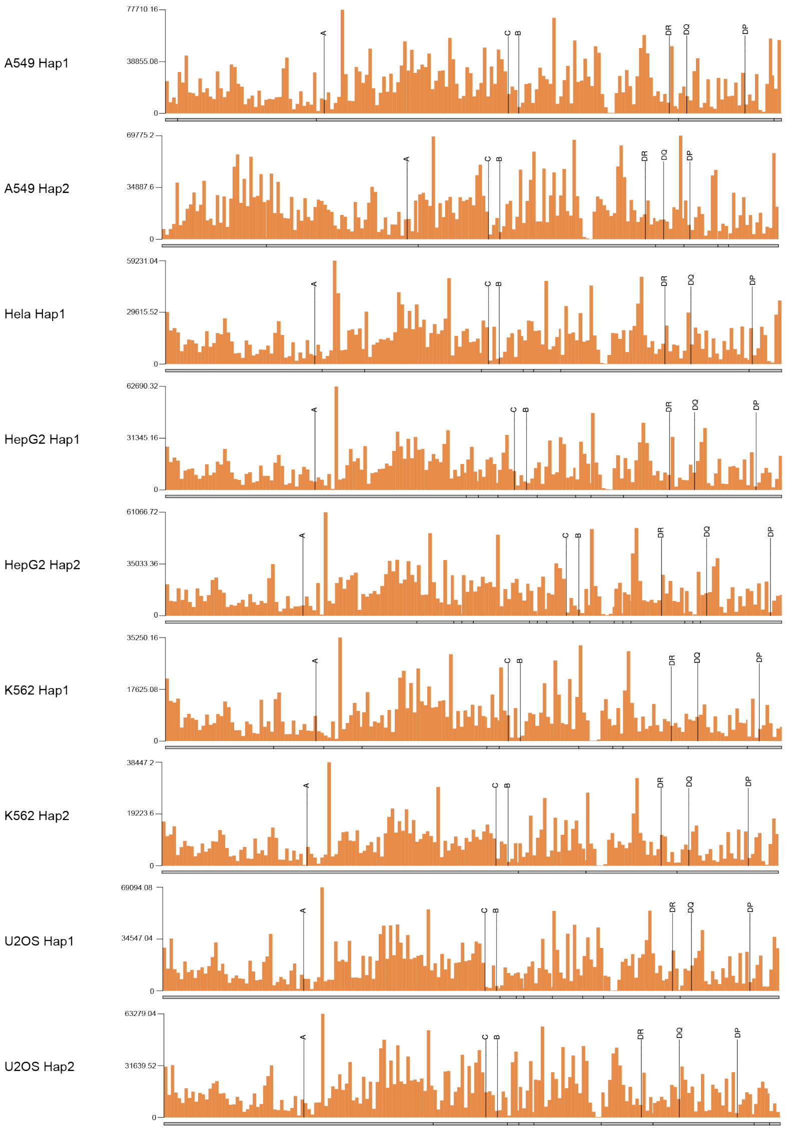

Figure S9

The HiFi reads coverage of the MHC region in the A549, Hela, HepG2, K562, and U2OS cell lines aligned to the assembled MHC haplotypes.
